# Circular to the Physicians of Kentucky

**Published:** 1855-05

**Authors:** S. D. Gross

**Affiliations:** Louisville


### CIRCULAR.

### TO THE PHYSICIANS OF KENTUCKY.

THE undersigned having been appointed by the Kentucky State Medical Society a Committee to report on the "Statistics of Lithotomy and the prevalence of Calculous Diseases in Kentucky," earnestly solicits information on the following subjects:—

1st. What has been the number of cases of stone in the bladder, urethra, and kidneys in your region of country, as observed by yourself personally, or reported to you by reliable authority?

2nd. Are there any calculous disorders, that is, gravel and urinary deposits, in your district, and if so, what are the most frequent varieties?

3rd. What influence, if any, do age, sex, color, occupation, food, drink, modes of life, and geological formation, exert upon the production of the above diseases?

4th. Have you any reason to believe that the use of lime-water, malt-liquors, cider, wine, ardent spirits, pork, corn bread, hot wheat bread, coffee, tea, and other articles of diet tend to favor the development of stone in the urinary organs and of calculous disorders.

5th. Is there reason to suppose that gout, rheumatism, intermittent fever, and other diseases exert any influence upon the production of these affections?

6th. How many operations have been performed for the removal of stone from the bladder in your region of country? Name the operators and their methods, together with the age, sex, color, occupation, and mode of life of the patient, the duration of the disease, and the results of the case; if successful, whether there was any relapse; if fatal, how soon, with the cause of death, and the post-mortem appearances. In either event, state the size, form, weight, and composition of the calculus.

7th. Have you noticed any cases of stone or gravel among the inferior animals?

As it is the intention of the undersigned to report at the next meeting of the Society, it is very desirable that the answers to the above queries should be sent as early as the 1st of October next. It is to be hoped that the numerous topics involved in them will be duly considered by every intelligent practitioner of the State. It is very important that the whole subject should be most thoroughly investigated; and it is obvious that this can be done, not by the efforts of a single individual, but by the united labors of many. Every physician of Kentucky, therefore, should consider himself under obligation to contribute whatever knowledge he may possess on the subject to the general stock. By ascertaining the etiology of these affections, we may, perhaps, ultimately counteract their formation, and thus prevent a vast amount of human suffering. Kentucky is the great centre of calculous diseases of the American Union; and no where, either in the New world or the Old, has the operation of lithotomy been performed with more brilliant success.

S. D. GROSS.

*Louisville*, May 15, 1855.

